# Development of a Fur-dependent and tightly regulated expression system in *Escherichia coli* for toxic protein synthesis

**DOI:** 10.1186/1472-6750-13-25

**Published:** 2013-03-19

**Authors:** Lingyu Guan, Qin Liu, Chao Li, Yuanxing Zhang

**Affiliations:** 1 State Key Laboratory of Bioreactor Engineering, East China University of Science and Technology, Shanghai, P. R. China

## Abstract

**Background:**

There is a continuous demanding for tightly regulated prokaryotic expression systems, which allow functional synthesis of toxic proteins in *Escherichia coli* for bioscience or biotechnology application. However, most of the current promoter options either are tightly repressed only with low protein production levels, or produce substantial protein but lacking of the necessary repression to avoid mutations initiated by leaky expression in the absence of inducer. The aim of this study was to develop a tightly regulated, relatively high-efficient expression vector in *E. coli* based on the principle of iron uptake system.

**Results:**

By using GFP as reporter, P_fhuA_ with the highest relative fluorescence units, but leaky expression, was screened from 23 iron-regulated promoter candidates. P_fhuA_ was repressed by ferric uptake regulator (Fur)-Fe^2+^ complex binding to Fur box locating at the promoter sequence. Otherwise, P_fhuA_ was activated without Fur-Fe^2+^ binding in the absence of iron. In order to improve the tightness of P_fhuA_ regulation for toxic gene expression, Fur box in promoter sequence and *fur* expression were refined through five different approaches. Eventually, through substituting *E. coli* consensus Fur box for original one of P_fhuA_, the induction ratio of modified P_fhuA_ (named P_fhuA1_) was improved from 3 to 101. Under the control of P_fhuA1_, strong toxic gene *E* was successfully expressed in high, middle, low copy-number vectors, and other two toxic proteins, Gef and MazF were functionally synthesized without *E. coli* death before induction.

**Conclusions:**

The features of easy control, tight regulation and relatively high efficiency were combined in the newly engineered P_fhuA1_. Under this promoter, the toxic genes *E*, *gef* and *mazF* were functionally expressed in *E. coli* induced by iron chelator in a tightly controllable way. This study provides a tightly regulated expression system that might enable the stable cloning, and functional synthesis of toxic proteins for their function study, bacterial programmed cell death in biological containment system and bacterial vector vaccine development.

## Background

With the advent of the post-genomic era coming, the need is boosting to express a growing number of genes originating from different organisms [1]. Unfortunately, many of these foreign genes severely interfere with the survival of *Escherichia coli* cells, which could lead to bacteria death or cause significant defects in bacteria growth. What’s more, following the development of biological technology, many genetically engineered *E. coli* have been constructed and developed for different purposes, such as bioremediation, biomedicine and bioenergy [2]. However, their practical applications in the field are still restricted because engineered bacteria may cause new environmental contaminations. To minimize the potential risks, biological containment system was designed to monitor and restrict the distribution of engineered bacteria. Generally, containment systems are based on a ‘killer gene’ and a tight ‘regulatory circuit’ that controls expression of the killer gene in response to the presence or absence of environmental signals [3,4]. In order to activate the killer gene expression only at expected condition, usually a specific environment, the promoter that controls bacteria programmed cell death needs to be easily controllable, tightly regulated and environment-responsive.

In general, there are five solutions to manage killer or highly toxic genes expression: manipulation of transcriptional and translational control elements of highly toxic genes, manipulation of the coding sequence, manipulation of the copy number, addition of stabilizing sequences, and empirical selection of *E. coli* strains [5]. Among these solutions, manipulation of transcriptional control elements aims at blocking the leaky expression from the common used promoters, such as P_lac_, P_trc_, P_tac_, P_T7_, P_pL_, P_tetA_ or P_lacUV5_, which is critical in toxic gene expression [6,7]. As a result, the manipulation strategies of transcriptional control elements have been described to reduce the possibility of a unexpected toxic event. The strategies include suppression of basal expression from leaky inducible promoters, suppression of read-through transcription from cryptic promoters, tight control of plasmid copy numbers and proteins production as inactive (but reversible) forms [5,8-10].

Since the incomplete repression of promoter presents a major problem when cloning genes that encode lethal product to the bacterial host [11], it is obviously critical to tighten the control of gene expression by utilizing a tightly controllable promoter that permits *E. coli* normal growth until the very moment of highly toxic gene induction. Starting from this point, several tightly regulated expression circuits have been developed, such as P_rha_, hybrid P_lac/ara-1_, and P_BAD_-based vectors [8]. The widely used P_BAD_ derivative expression systems have been verified as useful solutions for their stringentness in toxic protein production in *E. coli*. Expression is induced to high levels on media containing L-arabinose, and tightly shuts off on media with glucose but without L-arabinose, which shows more stringent regulation of target gene expression than other expression systems. However, some problems still exist in this system, such as few vectors available and catabolism repression by glucose [6].

Usually, environment-responsive promoters are commonly engineered in inducible expression system, such as promoters sensing pH, temperature, oxygen concentration or iron availability [12]. From iron-uptake systems that are evolved by bacteria growing in iron-limiting environment, many iron-related promoters have been reported [13-15]. The promoter from iron-uptake regulon is strongly repressed in iron rich conditions by Fur, but fully derepressed in absence of iron [16]. Usually, Fur protein complexing with ferrous irons binds with high affinity to the 19-bp inverted repeat consensus sequence known as the Fur box (GATAATGAT [A/T] ATCATTATC) in the relevant promoter area, which controls transcription of iron-responsive genes in microorganisms [17]. Fur inhibits transcription initiation by blocking the entry of RNA polymerase (RNAP) to the promoter. This ability is tightly dependent on the relative affinities of RNAP and Fur to binding sites in the DNA [17].

In this study, a novel tightly inducible expression system was developed as an alternative of the current ones. This system, designated p_Y_P_fhuA1_, was capable of extremely tight regulation and allowed cloning of genes encoding highly toxic products within various copy-number plasmids. In detail, using *E. coli* Top10 as bacterial host, the strong iron-regulated promoter P_fhuA_ was selected as the primary candidate. By modifying Fur box in promoter sequences and Fur repressor synthesis, the tightness of P_fhuA_ was decreased to varying degrees. Thereinto, P_fhuA1_, which could be strict repressed by excess iron and efficiently induced by iron chelators, was the ideal promoter candidate for toxic gene expression in *E. coli* host.

## Results

### Preliminary screening for iron-regulated promoters

**Figure 1:**
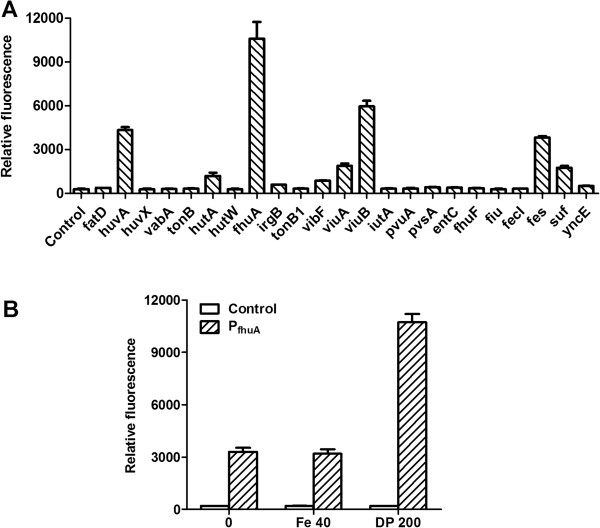
**GFP expressions under different promoters in iron-limitation (A), under P**_**fhuA **_**in variant iron concentrations (B). A.***E. coli* Top10 transformants harboring p_t_P_X_G were cultured in LB medium to OD_600_ = 0.8 and induced by 200 μM 2,2’-dipyridyl. Samples were taken at 20 h after induction for GFP assay; **B.** LB medium with 40 μM FeSO_4_ as iron-rich condition, LB medium with 200 μM 2,2’-dipyridyl as iron-limiting condition. The error bars represent the standard deviation (SD) for one independent experiment, performed in triplicate, the experiment was repeated for three times.

As described in the previous study [18], 23 promoter candidates were selected from *Vibrio parahaemolyticus*, *Vibrio cholerae*, *E. coli and Vibrio anguillarum*, and their transcription abilities were investigated with pUT_t_G as screening vector and iron chelator 2,2’-dipyridyl as inducer. Samples were adjusted to OD_600_ = 1 to measure the green fluorescence emitted by GFP, so the promoter strength and regulation could be simply correlated with the GFP fluorescence. As seen in Figure 1A, the promoters P_suf_, P_fes_, P_huvA_, P_fhuA_, P_viuB_, and P_viuA_ showed relatively high transcription activities with the relative fluorescence (RF) units of over 1,500 under iron-limiting medium. Among them, P_fhuA_ owned the highest RF units of 10595, and was chosen as the primary candidate for further study.

To evaluate the regulation performance of P_fhuA_, the GFP synthesis was detected when Top10/p_t_P_fhuA_G growing in LB medium supplemented with repressor (40 μM FeSO_4_) or inducer (200 μM 2,2’-dipyridyl). As shown in Figure 1B, even with the repressor addition, an obvious leaky expression was detected under P_fhuA_ transcription. Based on the data, the induction ratio that showed the tightness of promoter was calculated as 3.4, indicating that the tightness of P_fhuA_ had to be improved for the toxic gene expression.

### Modifications of P_fhuA_ to improve its tightness

**Figure 2:**
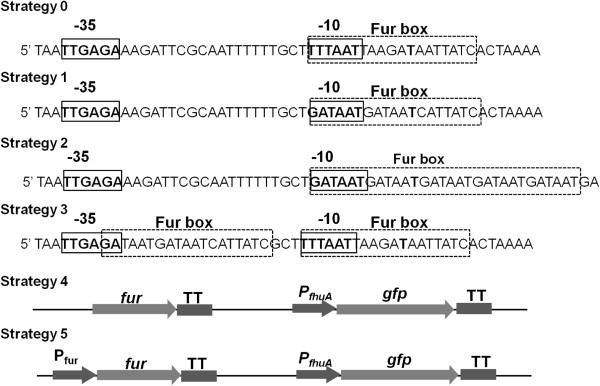
**P**_**fhuA **_**modified strategies used in this study.** Continuous line frame, -35 or −10 region; dash line frame, Fur binding box; P_fhuA_, *fhuA* promoter; TT, transcriptional terminator rrnBT1T2 derived from the rrnB rRNA operon of *E. coli*.

P_fhuA_ was from *V. cholerae* ferrichrome outer membrane receptor encoded gene *fhuA*[19]. Its characteristic regions were demonstrated as the same result by three online promoter prediction tools-Softberry, BDGP and SCOPE. The Fur box [20] spans across −10 region (Figure 2). To improve P_fhuA_ tightness, the Fur box sequences were remolded according to *E. coli* consensus Fur box sequence GATAATGAT[A/T]ATCATTATC [16]. As shown in Figure 2, Strategy 0 represented the original P_fhuA_ sequence. In Strategy 1, the original Fur box of P_fhuA_ was changed into *E. coli* consensus Fur box sequence by base substitution. In Strategy 2, Fur box was substituted by enhanced *E. coli* Fur box as described by Escolar *et al*. [17]. The enhanced Fur box contained five repeats of GATAAT motif to strengthen the Fur protein binding. In Strategy 3, two Fur boxes were used. One was the original one spanning −10 region, and the other one was *E. coli* consensus Fur box sequence across −35 region without changing the spacer distance between −35 and −10 regions. In Strategy 4, the *fur* fragment without P_fur_ was inserted into the upstream of P_fhuA_*gfp* TT cassette for more Fur protein synthesis. In Strategy 5, with the same aim, the *fur* fragment including P_fur_ was inserted. In theory, more Fur proteins would be synthesized in Strategy 5 than in Strategy 4. All the above-mentioned strategies aimed to enhance the affinity of Fur to the promoter and increase the transcription tightness sequentially.

All the remolded recombinant plasmids were transformed into *E. coli* Top10, resulting in Top10/p_t_P_fhuA_G (Strategy 0), Top10/p_t_P_fhuA1_G (Strategy 1), Top10/p_t_P_fhuA2_G (Strategy 2), Top10/p_t_P_fhuA3_G (Strategy 3), Top10/p_t_P_fhuA_GSDfur (Strategy 4), Top10/p_t_P_fhuA_GP_fur_SDfur (Strategy 5) to check the promoter regulation behaviors. As shown in Table 1, the relative fluorescence units under repression and induction were used to calculate the induction ratio and relative induction fold. The highest relative induction fold of 51 and the lowest induction ratio of 3 were obtained with P_fhuA_. By replacing the original Fur box with *E. coli* consensus Fur box in Strategy 1, the repression of P_fhuA_ was increased greatly from 3 to 101, but the induction was decreased by almost half of original value. The similar performances were also obtained in other strategies. Especially Strategy 2, 3, 5 displayed the high tightness under detection limit, but their relative induction folds were as low as 9, 3 and 12, respectively, which meant very limited transcription efficiency. As in Strategy 4, more GFPs were induced than in Strategy 5 following by less Fur protein synthesis, and showed the induction ratio of 108, and relative induction fold of 17. Under the same condition, Strategy 1 was comparable with the well-known tight regulated promoter P_BAD_ with the induction ratio of 126 and relative induction fold of 30. Taken together, there was a balance between the tightness and transcription efficiency, and the improvement of tightness would be accompanied by the decrease of transcription efficiency. Taking these two criteria into consideration, P_fhuA1_ in Strategy 1, performed similarly as P_BAD_, was designated as the final promoter candidate for the next experiments.

**Table 1:** Comparison of different promoter modified strategies

**Strategy**	**Modified strategies and descriptions**	**Repression RF**	**Induction RF**	**Induction ratio**	**Relative induction fold**
NC	*E. coli* Top10	200 ± 10	200 ± 9	0	1
PC	P_BAD_	260 ± 11	7812 ± 31	126	30
0	Original *fhuA* promoter	3292 ± 20	10116 ± 37	3	51
1	Changed the Fur box in −10 region into a conserved Fur box from *E. coli*[17]	250 ± 12	5466 ± 26	101	27
2	Modified the Fur box in −10 region into an enhanced Fur box [17]	196 ± 5	1885 ± 17	BDL	9
3	Integrated a designed Fur box in −35 region	200 ± 9	662 ± 19	BDL	3
4	Inserted the SD-fur-TT circuit in the vector	230 ± 9	3464 ± 24	108	17
5	Inserted the P_fur_-SDfur-TT circuit in the vector	195 ± 7	2389 ± 26	BDL	12

Repression RF = Relative fluorescence units under repression condition (LB + 40 μM FeSO_4_);

Induction RF = Relative fluorescence units under induction condition (LB + 200 μM 2,2’-dipyridyl);

Induction ratio = (Induction RF – NC’s Induction RF)/ (Repression RF- NC’s Repression RF), indicating the tightness of promoter;

Relative induction fold = Induction RF/NC’s Induction RF, indicating the transcription efficiency of promoter;

BDL, beyond detection limit; NC, Negative control; PC, Positive control.

### Performance evaluation of pP_fhuA1_ as an inducible tightly regulated expression system

**Figure 3:**
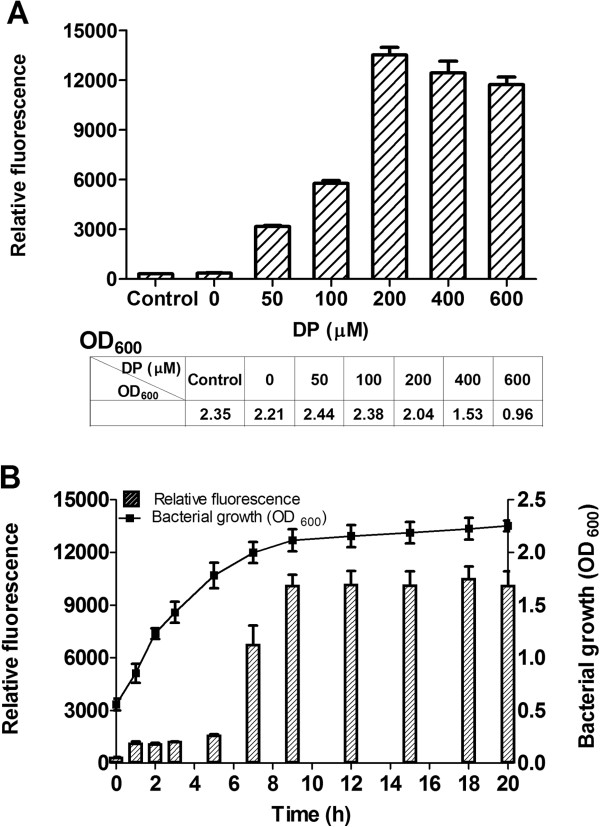
**P**_**fhuA1**_**-controlled GFP expression induced by 2,2’-dipyridyl. A**. GFP expression levels of *E. coli* Top10/p_t_P_fhuA1_ under the induction of different 2,2’-dipyridyl concentrations. OD_600_ in the table represented the OD_600_ value of cultures at 20 h post-induction. **B**. Time course analysis of GFP expression in *E. coli* Top10/p_t_P_fhuA1_ after induction with 200 μM 2,2’-dipyridyl. 2,2’-dipyridyl was added at 0 h. The error bars represent the standard deviation (SD) for one independent experiment, performed in triplicate, the experiment was repeated for three times.

The ability to modulate the target gene expression by partial induction of the promoter activity by applying different concentrations of inducer is a desirable feature for a controllable expression system. To investigate the possibility to modulate the P_fhuA1_-initiated target gene expression in an inducer concentration-dependent manner, the specific GFP yields were measured from the cells of transformed with p_t_P_fhuA1_G induced with various concentrations of 2,2’-dipyridyl (0, 50, 100, 200, 400, and 600 μM). In Figure 3A, an exponential GFP yields response was observed with 2,2’-dipyridyl in the range of 0 to 200 μM, which culminated in a wide range of GFP synthesis with the relative fluorescence units from 350 to 13520. The maximal specific GFP yield was obtained in 200 μM 2,2’-dipyridyl. However, as 2,2’-dipyridyl concentration increased to 400 and 600 μM, GFP yields decreased gradually. This could be attributed to the harsh iron-limiting condition created by high concentration of 2,2’-dipyridyl for chelating divalent cations, which impaired the *E. coli* growth for the difficulty to uptake enough iron from the medium. It could be found that 2,2’-dipyridyl less than 200 μM exerted little influence on *E. coli* growth, and the OD_600_ values at the end of induction were between 2.0 and 2.5. As 2,2’-dipyridyl concentration continued to increase to 400 μM and 600 μM, OD_600_ value drastically decreased to 1.5 and 1.0, respectively. Therefore, 0 ~ 200 μM 2,2’-dipyridyl could partially induce and 200 μM was the preferable inducer concentration to fully switch on p_t_P_fhuA1_ expression system and to minimize the harmful effect of the inducer on *E. coli* growth.

In order to see the time response of p_t_P_fhuA1_ by which it is effectively turned on, GFP yields along with *E. coli* growth were detected. GFP expression in the p_t_P_fhuA1_-bearing culture was continuously increased until 9 h post induction (Figure 3B). During the first 1 h induction, the relative fluorescence value increased by 3.4 folds, which meant the expression circuit had been switched on. Afterwards, the GFP expression increased very slowly from 1 to 5 h, and underwent a jump from 5 to 9 h. At last, the GFP expression achieved its peak at 9 h post induction, corresponding to the beginning of stationary phase in *E. coli* growth. Thus, the whole expression circle for P_fhuA1_ lasted for 9 h. In other side, the GFP synthesis always lagged behind the *E. coli* growth. This is desirable in toxic gene expression to reduce the damage of protein products to cells.

### Toxic protein expression under the control of modified P_fhuA1_

**Figure 4:**
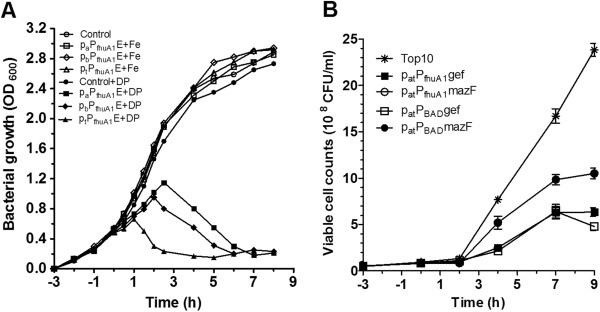
***E. coli *growth with toxic gene expression under the control of P**_**fhuA1**_**. A**. Growth curves to demonstrate the tightly controllable expression of lysis gene *E* in p_t_P_fhuA1_E, p_b_P_fhuA1_E and p_a_P_fhuA1_E under repression (+Fe, 40 μM FeSO_4_), or induction (+DP, 200 μM 2,2’dipyridyl). At −3 h, 40 μM FeSO_4_ was added; at 0 h, 200 μM 2,2’dipyridyl was added. Cultures were in duplicate. Bacterial growth was measured at OD_600_ nm. **B**. Colony forming units of Top10/p_at_P_fhuA1_gef and Top10/p_at_P_fhuA1_mazF after induction. At 0 h, 200 μM 2,2’dipyridyl was added. The error bars represent the standard deviation (SD) for three independent experiments, performed in triplicate.

Based on the tight regulatory control in GFP expression, the potential of p_Y_P_fhuA1_ in clone and expression of highly toxic gene products need to be verified. First, in order to check whether the basal expressions from P_fhuA1_ in different copy numbered vectors would allow the highly toxic protein synthesis in *E. coli*, pUT (pUC ori., high copy), pUT_b_ (pBBR1 ori., middle copy), and pUT_a_ (p15A ori., low copy) [21] were used as the back plasmids in toxic gene expression. Experimentally, toxic gene was inserted into the back plasmids with high-, middle- and low- copy number origins. Here the lysis gene of *E. coli* phage φX174, *E*, was selected as toxic gene, and its trace expression could induce lysis by formation of a transmembrane tunnel structure in the cell envelope of *E. coli*[22]. 200 μM 2,2’-dipyridyl as the optimal dose was added into LB medium for induction. As shown in Figure 4A, the similar growths of the strains bearing p_t_P_fhuA1_E, p_b_P_fhuA1_E and p_a_P_fhuA1_E were observed as the control strain in medium with repressor. Because of extreme sensibility of *E. coli* to E protein [22], the normal growth of these strains indicated the tight control of P_fhuA1_ activity in different copy-numbered vectors under the repression condition. However, under the induction conditions, their behaviors were diverse from each other. After Top10/p_t_P_fhuA1_E, Top10/p_b_P_fhuA1_E and Top10/p_a_P_fhuA1_E were induced at 0.48, 0.49 and 0.52 OD_600_, the highest OD_600_ (0.66, 0.95, and 1.15) was achieved 1, 2, and 2.5 h later, and the lowest OD_600_ (0.15, 0.21, and 0.23) appeared at 5, 6, and 7 h post induction, respectively. The differences of lysis performances caused by E protein production reflected the copy-number variant of vectors bearing P_fhuA1_. Sorting from fast to slow, although three strains with p_t_P_fhuA1_E, p_b_P_fhuA1_E, and p_a_P_fhuA1_E lysed in 5, 6, and 7 h, respectively, their lysis kinetics were in similar mode. This kind of typical lysis curves indicated that *E* expression was under a controllable promoter. As a result, P_fhuA1_ is tight enough to regulate high toxic gene expression in different copy-number vectors.

With the successful application of p_Y_P_fhuA1_ in the regulation of *E* gene expression, its broad availability was the next concern. Then, other two toxic proteins were synthesized using p_Y_P_fhuA1_ in *E. coli* host. The *gef* encoding Gef of *E. coli* belongs to the *hok* killer gene family in Gram-negative bacteria and its expression kills the cell from the inside by interfering with a vital function in the cell membrane [23]. The *mazF* from a toxin-antitoxin module *mazEF* specifies a stable toxin that cleaves mRNA at a specific site(s) which is responsible for programmed cell death in *E. coli*[24]. Here p_at_P_fhuA1_ was used, which is a middle-low-copy number vector with ~30 copies per cell [21]. Colony forming units (CFUs) of the transformants were detected in the presence of inducer (Figure 4B). Comparing with the negative control Top10, all the recombinant strains were grown similarly in the presence of iron (data are not shown), and no leaky expression phenotype was appeared. However, the growth of Top10/p_at_P_fhuA1_gef and Top10/p_at_P_fhuA1_mazF was repressed when cultured with 2,2’-dipyridyl as the result of *gef* and *mazF* expression. At 0 h, 2,2’-dipyridyl was added. During the first 2 h after induction, all three strains performed to adapt the iron-limiting condition and maintained in a similar density of 10^9^ CFU/ml. However, after 2 h, their growth behaviors were obviously different. Top10 strain grew fast to 2.4×10^9^ CFU/ml in exponential way until 9 h post induction. However, the strains Top10/p_at_P_fhuA1_gef and Top10/p_at_P_fhuA1_mazF grew exponentially until 7 h post induction and then kept at 6×10^8^ CFU/ml and 1×10^9^ CFU/ml, respectively. Therefore, the growth repression shown by recombinant strains revealed that toxic genes *gef* and *mazF* were functionally expressed after induction under the control of P_fhuA1_. Furthermore, p_Y_P_fhuA1_ could be applied in different toxic gene expressions.

## Discussion

**Figure 5:**
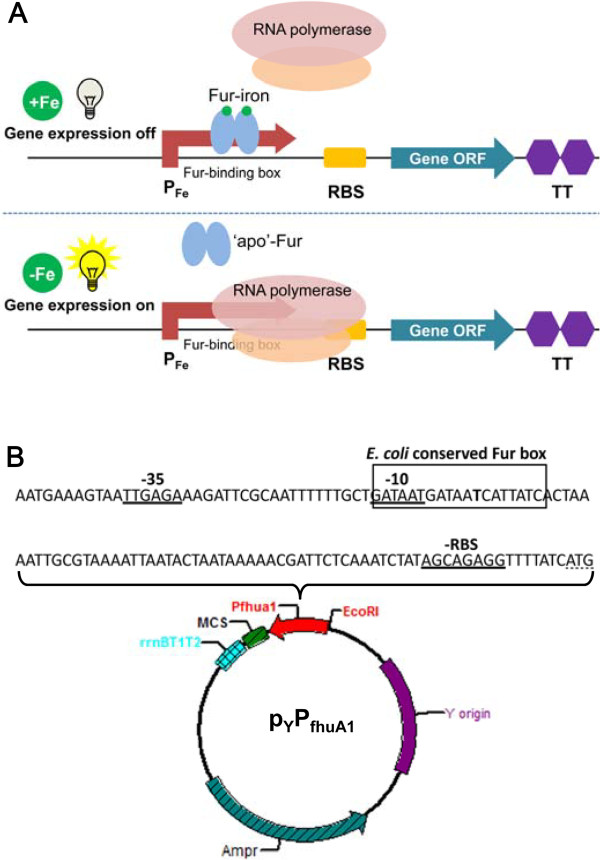
**A. A schematic diagram of the mechanism of tight Fur-dependent iron-limitation regulation switch developed for *E. coli *stains.** The mechanism is described in Results and Discussion. **B**. Physical map of the p_Y_P_fhuA1_ expression vector. Y, different replicate origins; MCS, multi-cloning sites; *rrnBT1T2*, ribosomal terminators T1 and T2.

The wide variety of applications of recombinant proteins and genetically engineered *E. coli* signifies the increasing demand for varied regulatory circuits. The challenges of regulatory expression technology are multifaceted to meet the growing need, in terms of quantities, qualities, cost-effectiveness and some particular cases. When even a low level of gene expression is detrimental to bacterial growth, the expression system has to be tightly regulated and efficiently shut off in the absence of inducer. In this work, a modified Fur-dependent and iron-limiting inducible expression system was constructed, which owns several prominent features and benefits that confers it an attractive and versatile expression system for highly toxic foreign protein synthesis. In this iron regulation system (Figure 5A), abundant iron complexing with Fur repressor binds to P_fhuA1_ sequence to prevent RNA polymerase contacting with the promoter region and to shut down the transcription. In the other side, without enough iron, Fur is disassociated from the Fur box of P_fhuA1_ sequence to open a place for RNA polymerase and to switch on the expression of downstream gene. This system is inducible by iron chelator addition. Most of all, the induction ratio was improved via enhancing the binding capacity between the repressor Fur and P_fhuA1_, which made the system was tight enough to express different kinds of lethal genes in *E. coli*. As shown in Figure 5B, the ultimate plasmid was named as p_Y_P_fhuA1_. According to different purposes of more or less recombinant protein needed, Y site could be replaced by high or low copy-numbered origin. Although the increased copy number of those vectors might cause higher levels of leaky expression, it was proved that high-copied p_t_P_fhuA1_ could successfully express highly toxic gene, such as *E* in *E. coli*. Besides, the exact P_fhuA1_ sequence and key regions of the plasmid were also shown. A strong *rrnBT1T2* terminator derived from the *rrnB* rRNA operon of *E. coli* located at downstream of multi-cloning sites. Taken together, when designing these vectors for toxic gene expression, the following properties had to be taken into account: a strong promoter capable of tightly regulation, strong terminators against read-through transcription from other plasmid-borne promoters, and different copy numbered origins of replication for gene expression with different degrees of toxicity. In all, this system is easily inducible, tightly controllable and potentially versatile for biotechnological application.

One of the most notable merits for this system is its tight regulation by iron-limitation signal. Escolar *et al*. once pointed out that the actual sequences recognized by Fur consisted of a minimal array of three conserved 6 bp (GATAAT) units which could be extended laterally by discrete additions of repeats of the same unit, thereby giving rise to new sites to which the repressor binds with a range of affinities [17]. It was assumed that the affinity would vary depending on both the number of repeats present on each operator and the conservation of their sequences, which would allow an entire range and hierarchy of transcription responses depending on small changes in the iron status in the cell [17]. Based on this hypothesis, we designed three types of Fur boxes differing in sequence conservation and 6-bp unit repeat number. It was found that the more repeats of conserved 6-bp units or the more conserved Fur box, the stronger affinity between Fur and the promoter, which was following by the tighter promoter. This verified the hypothesis mentioned above. In another side, this strategy also provides a new way for modifying other inducible promoters. However, a problem in this strategy is that the transcription efficiency decreases along with the tightness improvement. This is in common, because most of the regulatory systems have an intrinsic limitation in the range of induction, and attempts to mutate promoters to reduce basal expression usually result in concomitant reduction of induced levels. To overcome this drawback, Royo *et al*. used the *nasF* attenuator and the NasR-dependent anti-termination system from *Klebsiella oxytoca* to construct a novel expression circuit which could conditionally prevent undesired transcription from any transcriptional initiation signal, while keeping induced levels intact [10,25]. This point of view gives us a new reference for further optimization of expression vector constructed here.

Although iron is one of the most abundant elements on Earth, in aerobic environments it is predominantly found as ferric (hydro)oxides that are relatively insoluble at neutral pH, and thus, ionic ferric (Fe^3+^) concentrations are exceedingly low [26]. Therefore, as an iron limitation responsive vector, p_Y_P_fhuA1_ has great potential in developing ‘suicidal’ containment system for environmentally relevant application. Furthermore, in our previous research, iron-limiting condition was verified as an *in vivo* stimulating signal, and promoters derived from iron uptake system could be induced *in vivo*[18]. Therefore, this means P_fhuA1_ could be used in the construction of *in vivo* inducible antigen synthesis system, which could alleviate the toxicity or metabolic burden of the host strain and improve the immunogenicity for bacterial vector vaccine [27-32]. Moreover, *in vivo* regulated and delayed attenuation strategy has been developed for maintaining the invasive abilities of bacterial vector to the greatest extent, such that the recombinant vaccine has the ability as virulent wild-type strain to reach effector lymphoid tissues before display of attenuation to preclude onset of any disease symptoms [33]. p_Y_P_fhuA1_ could also be applied in construction of *in vivo* delayed attenuation vaccine. In all, p_Y_P_fhuA1_ could be induced by iron chelators in medium, *in vitro* aerobic environments and *in vivo* animal host, and has extensive application in biotechnology area.

## Conclusions

In all, as the expression vector particularly developed for toxic protein synthesis, p_Y_P_fhuA1_ owns its notable advantages, which include easy control, tight regulation, relatively high efficiency and multi-environments response potential. Consequently, p_Y_P_fhuA1_ has great potential in functional study of toxic genes, construction of biological containment system, or bacterial vector vaccine development.

## Methods

### Bacterial strains and growth conditions

The common clone vector *E. coli* Top10F’ (F’[*lac*I^q^ Tn*10* (Tet^R^)] *mcr*A Δ(*mrr*-*hsd*RMS-*mcr*BC) Φ80 *lac*Z ΔM15 Δ*lac*Χ74 *rec*A1 *ara*D139 Δ(*ara-leu*)7697 *gal*U *gal*K *rps*L (Str^R^) *end*A1 *nup*G, Invitrogen) was chosen as bacterial host. The plasmids used in this study are listed in Table 2. *E. coli* strains were grown at 37°C in lysogeny broth (LB) medium (1% tryptone, 0.5% yeast extract, 0.5% NaCl) [34]. When required, ampicillin (Amp, 100 μg/ml), FeSO_4_ (40 μM) and/or different concentrations of 2,2’-dipyridyl were added.

**Table 2:** Plasmids used in this study

**Plasmid**	**Description**	**Reference**
pUT	pUC18 derivative with *rrnBT1T2* terminator, Ap^r^	[18]
pUT_t_G	pUT derivative, with a reporter gene *gfp*, Ap^r^	[18]
pUT_b_	pUC18 derivative with *rrnBT1T2* terminator, pBBR1 ori., Ap^r^	[21]
pUT_a_	pUC18 derivative with *rrnBT1T2* terminator, p15A ori., Ap^r^	[21]
pUT_at_	pUC18 derivative with *rrnBT1T2* terminator, pAT153 ori., Ap^r^	[21]
p_t_P_fhuA_G	pUT derivative containing P_fhuA_*gfp* TT, Ap^r^	This study
p_t_P_fhuA1_G	pUT derivative containing P_fhuA1_*gfp* TT, Ap^r^	This study
p_t_P_fhuA2_G	pUT derivative containing P_fhuA2_*gfp* TT, Ap^r^	This study
p_t_P_fhuA3_G	pUT derivative containing P_fhuA3_*gfp* TT, Ap^r^	This study
p_t_P_fhuA_GSDfur	pUT derivative containing P_fhuA_*gfp* TT and SD-*fur* TT from *E. coli*, Ap^r^	This study
p_t_P_fhuA_GP_fur_SDfur	pUT derivative containing P_fhuA_*gfp* TT and P_fur_*fur* TT from *E. coli*, Ap^r^	This study
p_t_P_fhuA1_E	pUT derivative containing P_fhuA1_*E* TT, Ap^r^	This study
p_b_P_fhuA1_E	pUT_b_ derivative containing P_fhuA1_*E* TT, Ap^r^	This study
p_a_P_fhuA1_E	pUT_a_ derivative containing P_fhuA1_*E* TT, Ap^r^	This study
p_at_P_fhuA1_E	pUT_at_ derivative containing P_fhuA1_*E* TT, Ap^r^	This study
p_at_P_fhuA1_gef	pUT_at_ derivative containing P_fhuA1_*gef* TT, Ap^r^	This study
p_at_P_fhuA1_mazF	pUT_at_ derivative containing P_fhuA1_*mazF* TT, Ap^r^	This study

### Plasmid construction

A reporter plasmid pUT_t_G constructed previously was used as the promoter screening vector [18]. The primers from the former work were applied to amplify the candidate promoters from bacterium chromosomes or plasmids [18]. The amplified promoter products, possessing the native start codon, Shine-Dalgarno sequence, and −35 and −10 promoter elements plus additional upstream bases, were inserted into pUT_t_G, and the resultant plasmids were transformed into *E. coli* Top10 named Top10/p_t_P_X_G (_X_ mean different promoters) for iron-regulated promoter screening.

The primers listed in Table 3 were used to modify the P_fhuA_ promoter sequences and its regulation. P_fhuA1_ and P_fhuA3_ fragments were all amplified from the original P_fhuA_ sequence in the previous work [18], and P_fhuA2_ (TAATGAAAGTAATTGAGAAAGATTCGCAATTTTTTGCTGATAATGATAATGATAATGATAATGATAATGAAAAATTAATACTAATAAAAACGATTCTCAA) was synthesized by Life Technologies (Shanghai, China). The three promoter fragments fused with their relevant GFPs by overlap polymerase chain reaction (PCR), respectively. The resultant fragment was ligated into *Pvu*II/*EcoR*I digested plasmid p_t_P_fhuA_G. The 276 bp *E* gene was amplified from PhiX174 genome, and the amplification of 153 bp *gef*, 336 bp *mazF* fragments were used *E. coli* DH5α (F^-^,φ80d*lac*ZΔM15, Δ(*lac*ZYA-*arg*F)U169, *deo*R, *rec*A1, *end*A1, *hsd*R17(r_k_^-^,m_k_^+^), *pho*A, *sup*E44, λ-, *thi*-1, *gyr*A96, *rel*A1, Invitrogen) chromosome as templates. The modified P_fhuA1_ was inserted into *EcoR*I/*BamH*I sites of pUT, pUT_b_, pUTa and pUT_at_, and then the resultant plasmids were ligated with *BamH*I/*Pst*I digested toxic genes *E*, *gef*, *mazF* to get recombinant plasmids p_t_P_fhuA1_E, p_b_P_fhuA1_E, p_a_P_fhuA1_E, p_at_P_fhuA1_gef and p_at_P_fhuA1_mazF (Table 2). At last, all the recombinant plasmids were transformed into *E. coli* Top10 for P_fhuA_ regulation test.

**Table 3:** PCR primers used in this study

**Name**	**Sequence (5’-3’)**^**a**^
P_FhuA1_-for	CGGAATTCCAGTACCAGTGCCGCCATCGTCCA
P_FhuA1_-rev	TGATTATCATTATCAGCAAAAAATTGCGAATCTTTCTC
GFP1-for	ATAATGATAATCATTATCACTAAAATTGCGTAAAATTAAT
GFP1-rev	AAAACTGCAGTTATTTGTACAGTTCATCCATGCCA
P_FhuA2_-for	CGGAATTCCCTTAATGAAAGTAATTGAGAAAGAT
GFP2-for	AACGATTCTCAAATCTATAGCAGAGGTTTTATCAT
GFP2-rev	AAAACTGCAGTTATTTGTACAGTTCATCCATGC
P_FhuA3_-for	CGGAATTCACCAGTACCAGTGCCGCCATCGTC
P_FhuA3_-rev	TAATGATTATCATTATCTCAATTACTTTCATTAAGGCACC
GFP3-for	ATGATAATCATTATCGCTTTTAATTAAGATAATTATCACT
GFP3-rev	AAAACTGCAGTTATTTGTACAGTTCATCCATGCCAT
Fur-for	CAGCTGGAGCAAATTCTGTCACTTCTTCTA
Fur-rev	CGGAATTCATAAGTGAGAGCTGTAACTCTCGC
P_Fur_-for	CAGCTGCTGGCTGATGATGACCACTTTGT
P_Fur_-rev	CGGAATTCATAAGTGAGAGCTGTAACTCTCGC
E-for	CGCGGATCCATGGTACGCTGGACTTTGTGGGA
E-rev	AAAACTGCAGTCACTCCTTCCGCACGTAATTTT
gef-for	CGCGGATCCATGAAGCAGCATAAGGCGATGATTG
gef-rev	AAAACTGCAGTTACTCGGATTCGTAAGCCGTGAAA
mazF-for	CGCGGATCCATGGTAAGCCGATACGTACCCGATAT
mazF-rev	AAAACTGCAGCTACCCAATCAGTACGTTAATTTT

^a^ Restriction enzyme sites used for cloning of PCR products are underlined.

### General DNA procedure and online analysis tools

General DNA operations were carried out following the standard protocols. Automated DNA sequencing and primer synthesis were completed by Life Technologies (Shanghai, China). P_fhuA_ characteristic regions were predicted by online tools: BPROM-bacterial promoter prediction program from Softberry (http://linux1.softberry.com/berry.phtml?topic=bprom&group=programs&subgroup=gfindb), BDGP-Promoter (http://www.fruitfly.org/seq_tools/promoter.html) and SCOPE-Suite for Computational identification Of Promoter Elements [35].

### GFP synthesis detection

Overnight cell cultures were inoculated (1:100, v/v) into fresh LB medium containing appropriate antibiotics and cultured in a shaker at 200 rpm and 37°C. At middle log phase typically with an optical density at OD_600_ = 0.8-1.0, 2, 2’-dipyridyl was added to induce the expression of GFP. After 20 hours or defined time points of iron-limiting induction, 1 ml cell culture sample was taken, centrifuged at 12,000 *g* for 3 min, washed and resuspended in PBS (pH 7.2) to the same OD_600_ value (OD_600_ = 1.0). For each sample, 100 μl of cell suspension was added into a 96-well flat-bottom polystyrene plate (Costar, USA) and measured with a fluorescence plate reader (TECAN, GENios Pro, Austria). Excitation wavelength was set at 485 nm and emission was detected at 535 nm.

### Toxic gene expression detection

The *E. coli* strains Top10/p_t_P_fhuA1_E, Top10/p_b_P_fhuA1_E, Top10/p_a_P_fhuA1_E, Top10/p_at_P_fhuA1_gef and Top10/p_at_P_fhuA1_mazF were overnight grown at 37°C in LB medium supplemented with 40 μM FeSO_4_ to ensure tight repression of toxic gene. To induce toxic gene expression, the culture was diluted to an OD_600_ of 0.1 and cultured in a shaker at 200 rpm and 37°C. At early log phase (OD_600_ = 0.3-0.4), 2,2’-dipyridyl was added into culture to create iron-limiting condition. Cell samples were taken at different time points after induction to measure both the OD_600_ and the colony formation units (CFUs) to determine the bacteria growth in iron-limiting medium.

## Competing interests

The authors declare that they have no competing interests.

## Authors’ contributions

LG participated in the design of the study, carried out the cloning studies, performed the statistical analysis and drafted the manuscript. QL conceived of the study, helped to draft the manuscript and coordinated the study. CL carried out the molecular genetics and expression studies. YZ helped to draft the manuscript. All authors read and approved the final manuscript.
